# Ferroptosis: mechanisms and links with diseases

**DOI:** 10.1038/s41392-020-00428-9

**Published:** 2021-02-03

**Authors:** Hong-fa Yan, Ting Zou, Qing-zhang Tuo, Shuo Xu, Hua Li, Abdel Ali Belaidi, Peng Lei

**Affiliations:** 1 Department of Neurology and State Key Laboratory of Biotherapy, West China Hospital, Sichuan University, and Collaborative Center for Biotherapy, 610041 Chengdu, China; 2 West China School of Basic Medical Sciences and Forensic Medicine, Sichuan University, 610041 Chengdu, China; 3 Melbourne Dementia Research Centre and the Florey Institute of Neuroscience and Mental Health, The University of Melbourne, Parkville, VIC 3052 Australia

**Keywords:** Cell biology, Chemical biology

## Abstract

Ferroptosis is an iron-dependent cell death, which is different from apoptosis, necrosis, autophagy, and other forms of cell death. The process of ferroptotic cell death is defined by the accumulation of lethal lipid species derived from the peroxidation of lipids, which can be prevented by iron chelators (e.g., deferiprone, deferoxamine) and small lipophilic antioxidants (e.g., ferrostatin, liproxstatin). This review summarizes current knowledge about the regulatory mechanism of ferroptosis and its association with several pathways, including iron, lipid, and cysteine metabolism. We have further discussed the contribution of ferroptosis to the pathogenesis of several diseases such as cancer, ischemia/reperfusion, and various neurodegenerative diseases (e.g., Alzheimer’s disease and Parkinson’s disease), and evaluated the therapeutic applications of ferroptosis inhibitors in clinics.

## Introduction

Ferroptosis is a newly identified iron-dependent cell death that is different from other cell death forms, including apoptosis and necrosis. The program involves three primary metabolisms involving thiol, lipid, and iron, leading to an iron-dependent generation of lipid peroxidation and, ultimately, cell death. Ferroptosis can be prevented by the enzymatic reaction of two major antioxidant systems involving glutathione peroxidase 4 (GPx4) that catalyzes the reduction of lipid peroxides in a glutathione-dependent reaction and the recently identified ferroptosis suppressor protein (FSP1) that catalyzes the regeneration of ubiquinone (Coenzyme Q10, CoQ10), which act as a lipid peroxyl radical trap.^1,2^ Specific inhibitors can prevent ferroptosis, e.g., ferrostatin-1 acts as a radical-trapping antioxidant (RTA).^1^

Ferroptotic cell death is accompanied by a series of variations in cell morphology, metabolism, and protein expression that allows discrimination from other forms of cell death. At the cellular and subcellular levels, cells undergoing ferroptosis adopt a characteristic rounded shape before cell death similar to necrotic cells, but there is no cytoplasmic and organelle swelling, or plasma membrane rupture.^1,3^ The nuclei in ferroptotic cells conserve its structural integrity, without condensation, chromatin margination, plasma membrane blebbing, or formation of apoptotic bodies,^1^ which are characteristic features of apoptosis.^4^ Also, morphological features such as double-membrane enclosed vesicles from autophagic cells and intensive blebbing and loss of plasma membrane integrity shown in pyroptosis, are not observed in ferroptotic cells.^5^ The lone distinctive morphological feature is mitochondria that appeared smaller than normal with increased membrane density.^1^

Ferroptosis is regulated by a set of genes and shows a variety of metabolic changes. The detection of these changes, as evidence of ferroptosis, is essential for further research. Iron is an essential part of driving intracellular lipid peroxidation and ferroptosis.^6^ Ferroptosis can be prevented by using iron chelators (e.g., deferoxamine), whereas supplying exogenous iron (e.g., ferric ammonium citrate) enhances ferroptosis.^1,7^ Several studies have shown that the regulation of genes related to iron metabolism can also regulate ferroptotic cell death, such as transferrin, nitrogen fixation 1 (NFS1), iron response element-binding protein 2 (IREB2), Nuclear receptor coactivator 4 (NCOA4), etc.^1,8,9^ So, iron abundance is an essential indicator for monitoring ferroptosis. FRET Iron Probe 1 (FIP-1), a fluorescence probe, is widely used to detect the change of labile iron status during ferroptosis.^10^ Also, iron concentration can be measured with inductively coupled plasma-MS (ICP-MS) or Perls’ Prussian Blue staining.^5^ Lipid peroxidation level is also one of the most critical indicators of ferroptosis.^1^ A significant increase in peroxidized phospholipids was observed in many ferroptosis models.^11,12^ BODIPY-C11 (or C11-BODIPY) and LiperFluo are currently two major assays used to measure lipid peroxidation in ferroptosis, while the latter is considered to be a more reliable probe owing to its higher specificity.^11^ Also, changes in GPx4 activity can be used as an indicator for ferroptosis, which can be monitored by either the nicotinamide adenine dinucleotide phosphate (NADPH) activity assay^13^ or quantification of phosphatidylcholine hydroperoxide with LC-MS.^5^

## The discovery of ferroptosis

**Fig. 1:**
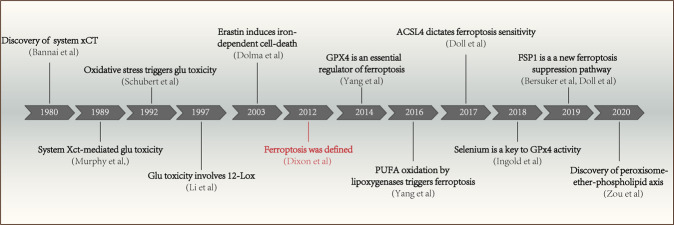
Timeline diagram depicting essential discoveries in the field of ferroptosis research. The discovery of ferroptosis starts with identification of system xCT, which was published in 1980. However, the term ‘ferroptosis' was only named in 2012.

Ferroptosis inducers were discovered in another high-throughput small molecule-screening study, as selectively lethal compounds to RAS mutant tumor cells, before the notion of ferroptosis was developed (Fig. 1). Back in 2003, erastin was found to be lethal with the expression of the engineered mutant Ras oncogene in human foreskin fibroblasts (BJeLR cells).^14^ However, subsequent studies have not identified sufficient targets for erastin-induced cell death.^3^ Ras-selective lethal small molecule (RSL)-3 and RSL5 were later identified in 2008 as synthetic compounds that selectively killed BJeLR cells in a non-apoptotic manner.^7^ It was not until 2012 that the form of cell death was named ferroptosis and erastin was found to inhibit cystine uptake by the cystine/glutamate antiporter (system ${\mathrm{X}}_c^ -$) leading to cell death (Fig. 1).^1^

System${\mathrm{X}}_c^ -$ was found to function by transporting cystine into the cell in exchange for glutamate in 1980.^15^ An early report has indicated that glutamate toxicity in a neuronal cell line is triggered by inhibition of cystine transport, leading to oxidative stress.^16^ Subsequently, it was discovered that antioxidant supplementation (e.g., alpha-tocopherol, α-toc) prevented glutamate-induced cell death in neuronal cell lines lacking N-methyl-D-aspartate receptor.^17^ Soon thereafter, it was shown that inhibition of arachidonate 12-lipoxygenase (Alox12), an iron-containing lipid dioxygenase, effectively inhibited cell death induced by glutamate in the hippocampal cell line HT22 and primary cortical neurons.^18^ Treatment of cells with exogenous arachidonic acid (AA), an Alox12 substrate, further accelerated cell death.^18^

In 2014, Yang et al. suggested that GPx4 plays a crucial role in protecting against ferroptosis by reducing phospholipid hydroperoxide and hence repressing lipoxygenase-mediated lipid peroxidation.^13^ In the extracellular milieu, the iron-carrier protein transferrin and glutamine were identified as essential factors required to induce ferroptosis. Conversely, inhibition of glutaminolysis and cell surface transferrin receptor can reduce heart injury triggered by ischemia/reperfusion (I/R) against ferroptosis.^19^ On the oxidation pathway, lipoxygenases (Lox) catalyze PUFA oxidation via a phosphorylase kinase G2 (PHKG2)-dependent iron pool,^19^ whereas compounds with RTA activity ameliorate ferroptosis via blocking lipid autoxidation.^20^

In 2017, it was shown that acyl-CoA synthetase long-chain family member 4 (ACSL4) is a biomarker and critical contributor to ferroptosis, that is required for the production of polyunsaturated fatty acids (PUFA) required for the execution of ferroptosis.^21^ A further study by Ingold et al. (2018), depicted the requirement for selenium utilization by GPx4 to inhibit ferroptosis.^22^ Recently, a new ferroptosis suppression pathway has been identified with the discovery that FSP1, CoQ10 oxidoreductase, can inhibit ferroptosis in a glutathione-independent pattern.^2,23^ In a further study of the ferroptosis sensitivity gene via genome-wide CRISPR–Cas9 suppressor screens, oxidative organelles peroxisomes were found to help the cancer cells escape and increase the susceptibility to ferroptosis through synthesizing polyunsaturated ether phospholipids (PUFA-ePL).^24^

## Regulation of ferroptosis

### Oxidation mechanisms

#### Summary of polyunsaturated fatty acids

Fatty acids are essential components of cellular lipid metabolism and fulfill several cellular functions, including energy supply, cell membrane formation, and serve as a precursor for several signaling molecules.^25^ However, tight regulation of fatty acid metabolism is required to prevent toxicity as observed in cell death pathways such as pyroptosis^26^ and ferroptosis. AMP-activated protein kinase (AMPK), a sensor of cellular energy status, can regulate ferroptosis via AMPK-mediated phosphorylation of acetyl-CoA carboxylase (ACC) and polyunsaturated fatty acid biosynthesis.^27^ Liver kinase B1 (LKB1) is a main upstream kinase responsible for the activation of AMPK in response to energy stress. The depletion of LKB1 also can sensitize mouse embryonic fibroblasts to lipid hydroperoxidation and ferroptosis.^28^ Long-chain fatty acids are mainly obtained from the diet and are named PUFA when they include more than two double bonds.^29^

PUFAs are components of the cell membrane and regulate several biological functions, including inflammation, immunity, synaptic plasticity, and cellular growth.^30^ The structure of PUFA is prone to oxidation because of the weak C–H bond at the bis-allylic positions.^31^ Furthermore, membrane PUFA is the primary target of reactive oxygen species (ROS) attack.^32^ In general, a higher number of double bonds in PUFA increases its susceptibility to oxidation.^33^ After the initial oxidation step, the free radicals can shift within the same molecule or oxidize further molecules.^34^ Therefore, PUFAs are the main substrate of lipid peroxidation during ferroptosis. Exogenous administration of the monounsaturated fatty acid (MUFA) oleic acid (OA, C18:1) can effectively inhibit erastin induced ferroptosis by competing with PUFAs for incorporation into phospholipids (PLs).^35^ This fact suggests that MUFAs are not the substrate of lipid peroxidation during ferroptosis. In addition, sterol lipids, including cholesterols, can be oxidized in membranes or low-density lipoprotein particles,^36^ and oxidized cholesterol is also the active substrate of GPx4.^37^ However, exogenous cholesterol treatment is not enough to regulate the lethality of RSL3 in human cancer cells. All evidence highlights the critical role of PUFA in ferroptosis.^38^

Research on PUFA mainly focuses on ω-6 and ω-3 eicosanoid.^30^ In vivo, the most common PUFA is AA, which is present in all tissues.^39^ While the composition of PUFA changes with the environment in many tissues, docosahexaenoic acid (DHA) and AA are the most abundant isoform of PUFA in the brain and retina,^40^ and DHA supplementation in childhood may improve cognitive and motor function in children with attention deficit/hyperactivity disorder.^41^ Due to its high lipid composition, the brain is particularly vulnerable to oxidative damage through lipid ROS. Therefore long-chain PUFA (LCPUFA) plays also an essential role in neurocognitive disorder diseases.^42,43^

#### The process of lipid peroxidation

**Fig. 2:**
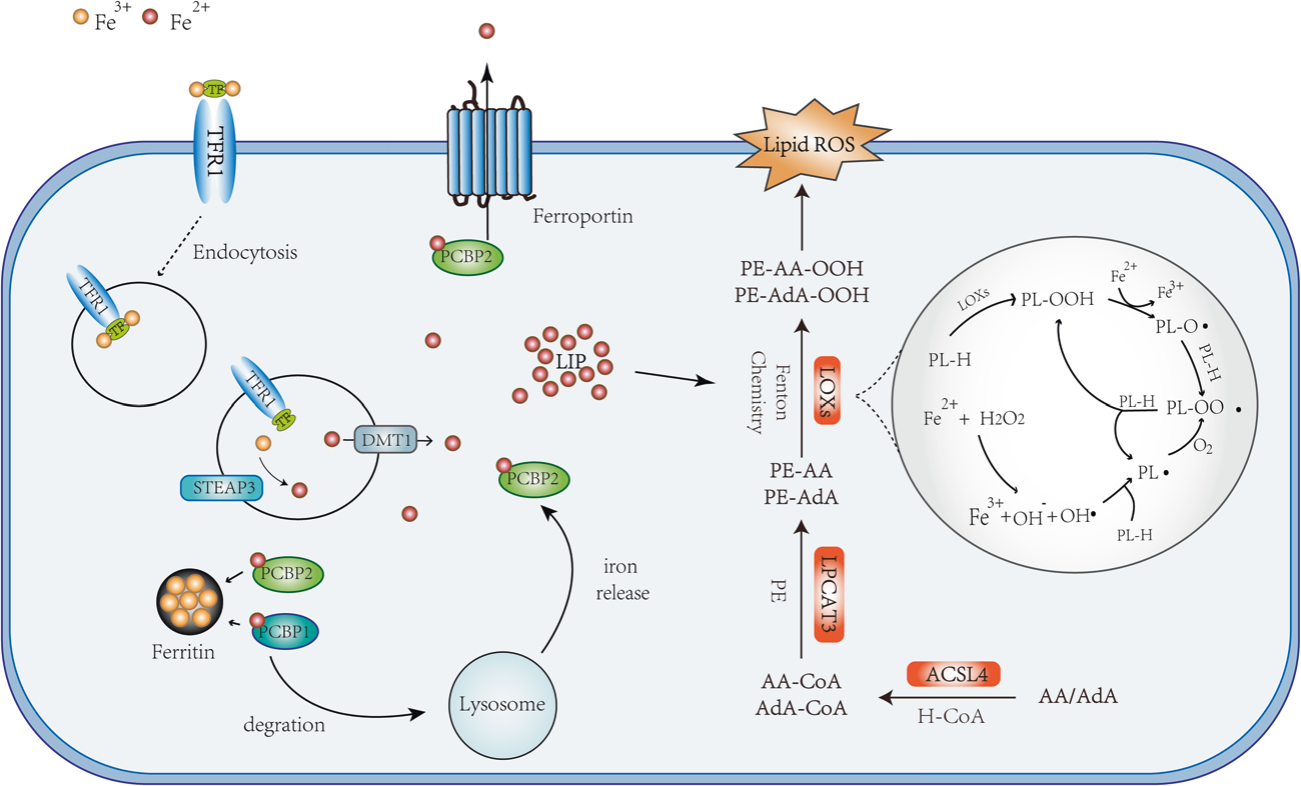
Schematic description of the signaling pathway of ferroptosis. The indicated pathways control ferroptosis sensitivity via lipid ROS generation. Phosphatidylethanolamines (PE); phospholipid (PL-H); phospholipid alkoxyl radical (PL-O·); phospholipid peroxyl radical (PL-OO·); phospholipid hydroperoxide (PL-OOH); transferrin (TF). The symbols used in the figure have been marked with names of the biomolecules.

PUFA is a double-edged sword, and its peroxidation may cause damage to cells. It can be integrated into the membrane by ACLS4^21^ and lysophosphatidylcholine acyltransferase 3 (LPCAT3).^44^ PUFA oxidation can occur either by non-enzymatic free radical chain reaction or enzyme catalysis (Fig. 2).

AA and adrenic acid (AdA) are the main PUFAs to induce ferroptosis.^11^ Taking AA as an example, ACSL4 catalyzes the ligation of CoA into AA to form a CoA-AA intermediate, which is esterified into phosphatidylethanolamine by LPCAT3 to form arachidonic acid-phosphatidylethanolamines (PE-AA). The oxidation of the formed PE-AA may follow either enzymatically through the action of Lox, or non-enzymatically through autoxidation to form PE-AA-OOH, both of which ultimately causes cell death.^1,38,45,46^ It has been reported that this reaction may occur on the mitochondrial membrane^1^ or the mitochondrial and endoplasmic reticulum membrane.^11^ BID (a pro-apoptotic protein) links mitochondria with ferroptosis,^47^ and the mitochondrial TCA cycle promotes ferroptosis.^48^

There are several hypotheses about the mechanism of ferroptotic cell death caused by lipid peroxides. At the structural level, PUFAs act as critical components of the cell membranes, and the extensive lipid peroxidation might transform the chemical and geometric structures of the lipid bilayer. Also, the accumulation of peroxidative lipid leads to membrane pores formation and destroys the barrier function, resulting in membrane thickness decrease and the change of membrane permeabilization.^49^ In a molecular dynamics study, lipid peroxidation increased the curvature of bio-membranes and acyl tails of peroxidative lipids, which are more hydrophilic, would bend to the water phase, causing membrane instability and micelle formation.^49^ These changes will eventually affect cell survival through inducing permeabilization. Lipid peroxides may be decomposed into toxic derivatives such as 4-hydroxynonenal (4-HNEs) and malondialdehyde (MDA). These by-products are produced by the decomposition of AA and other PUFAs through enzymatic and nonenzymatic pathways.^50^ 4-HNE and MDA were reported as the major toxic products which could react with DNA bases, proteins, and other nucleophilic molecules leading to serious cytotoxicity.^51^ Besides, once lipid peroxides are formed, they may further amplify ROS signaling and drive the mitochondrial caspase signaling pathway observed in pyroptosis,^52^ suggesting a potential link between ferroptosis and pyroptosis.

In this pathway, it is ACSL4 but not other ACSLs that changes the sensitivity of cells to ferroptosis by affecting the lipid composition,^21^ and ACSL4 reduction enhances resistance to ferroptotic cell death.^53^ I/R injury, ionizing radiation, and the inhibition of the NF2-YAP pathway can promote ferroptosis by rising ACSL4 expression.^54–56^ On the contrary, integrin α6β4 can mediate the activation of Src and STAT3, resulting in decreased expression of ACSL4 and suppression of ferroptosis.^57^

LPCAT3 is the most abundant subtype of acyltransferase and participates in transferring PUFA to the sn-2 position of cell membranes.^45,58^ The primary target of LPCAT3 is acetylated AA, which is inserted into membrane PLs in RSL3-induced ferroptosis.^44^ Liver X receptor can promote the expression of LPCAT3, facilitate the binding between AA and PLs, and increase the abundance of polyunsaturated phospholipids.^59^

#### The role of iron

Transferrin is the primary protein responsible for iron transport.^60^ The iron import starts by binding of iron-bound transferrin (Fe^3+^) to transferrin receptor 1 (TFR1, recently introduced as a specific ferroptosis marker^61^) and subsequent endocytosis in endosomes. In acidic endosomes, Fe^3+^ is reduced to Fe^2+^ by six-transmembrane epithelial antigens of the prostate 3 (STEAP3), and transported to the cytoplasm through divalent metal transporter 1 (DMT1).^62^ Cytosolic and mitochondrial labile iron pool (LIP), the intracellular nonprotein-bound redox-active iron, that can be used in cellular processes or stored into ferritin in a process mediated by the chaperones: Poly-(rC)-binding protein 1 (PCBP1) and PCBP2 to ferritin.^63^ Ferroportin (FPN) is the only known protein that exports intracellular iron in mammals,^64^ and the iron homeostasis is severely disturbed in FPN-deficient mice.^65^

The imbalance between iron import, storage, and export, may affect the cell susceptibility to ferroptosis. It has been shown that increased expression of transferrin receptor, induced by pseudolaric acid B, reinforces iron import and then triggers ferroptosis in glioma cells.^66^ Enhanced ferritin degradation in a process termed ferritinophagy, could increase the level of LIP and enhance ferroptosis.^67^ Recently, the Prominin2 protein has been shown to enhance ferroptosis resistance by promoting ferritin export.^68^ Iron and iron derivatives, such as heme or [Fe-S] clusters, are the essential active centers of many enzymes that are involved in ROS generation (Lipoxygenases, cytochrome P450, NADPH oxidases et al.).^6^

Electrons may escape from oxidation-reduction reaction and be captured by O_2_ to form superoxide (O_2_•), peroxides (H_2_O_2_ and ROOH), and free radicals (HO• and RO•).^69^ The oxidation by Fe^2+^ and H_2_O_2_, which is called Fenton reaction, would provide hydroxyl radicals that subtract hydrogen (H) from lipid to form a lipid radical (L•) as the start of the non-enzymatic reaction of lipid peroxidation.^70^ Lipid radicals combine with O_2_ to form lipid peroxyl radical (LOO•), which then snatches hydrogen from adjacent PUFA to form LOOH and a new lipid radical, and develops another oxidation reaction^71^(Fig. 2).

#### The role of lipoxygenases

Lox, a dioxygenase containing non-heme iron, catalyzes the oxidation of PUFA (with a 1-cis,4-cis-pentadiene structure) via stereospecific peroxidation,^72^ and the nomenclature of different Lox accounts for the specific site of their oxygenation product.^73^ There are six Lox isoforms in humans: 15-Lox-1, 15-Lox-2, 12-Lox-1, 12-Lox-2, E3-Lox, and 5-Lox, of which 12/15-Lox are widely distributed in different tissues.^74^ For tumor protein 53 (p53)-dependent cancer suppression,12-Lox-induced ferroptosis is crucial.^75^ 15-Lox selectively catalyzes PE-AA oxidation and executes ferroptotic cell death.^76^ The classical substrate of Lox is PUFA, and the sn2-15-hydroperoxy-eicasotetraenoyl-phosphatidylethanolamines (sn2-15-HpETE-PE) catalyzed by 15-Lox can be used as a signal of ferroptosis.^77^ Whereas, phosphatidylethanolamine-binding protein 1 (PEBP1), a scaffold protein inhibitor of protein kinase cascade, combines with 15-Lox after it is dissociated from RAF1 kinase, shows high selectivity and specificity for ETE-PE and promotes ferroptosis by generation of lipid death signals.^12^ NO•, a reactive free radical, was found to interact with other free radicals, disturb the lipid peroxidation caused by 15-Lox, and lead to the oxidative truncation of 15-HpETE.^78^

However, the key role of Lox in ferroptosis is still in debate. The preferred substrate of Lox is free PUFAs, so the first step for Lox is to cleave PUFA acyl chains from PLs through the activity of phospholipase.^79^ However, this model is inconsistent with ferroptosis, where the lipid peroxidation in ferroptosis occurs on esterified PUFA-PLs rather than free type, implicated by the role of LPCAT3 in ferroptosis^44,58^ and the facts as mentioned earlier that the MUFA OA strongly suppresses erastin-induced ferroptosis by competing with PUFAs for incorporation into PLs.^35^ In some studies, 12/15-Lox deletion cannot rescue the embryonic lethality of GPx4 knockout mice, nor can it eliminate the cell death following whole-body GPx4 deletion in adult mice.^80,81^ In addition, some cell lines sensitive to ferroptosis did not express any major Lox enzyme.^82^ Therefore, Lox may not be necessary in ferroptosis, or it may play a role in some more complex environments or situations by complementing the autoxidation pathway, which should be further investigated.

### Antioxidant mechanisms

#### The GPx4 pathway

**Fig. 3:**
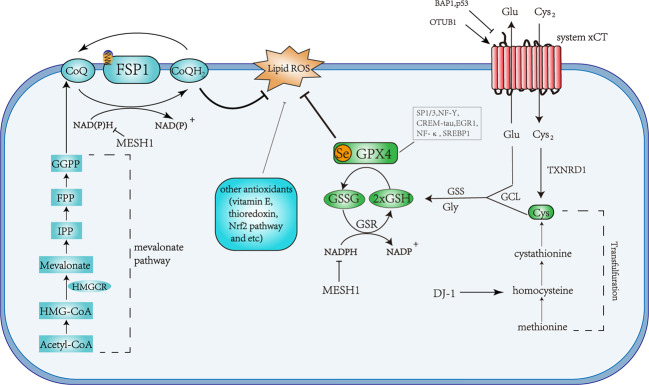
Overview of the anti-ferroptosis pathway. Schematic description of the two defense mechanisms identified in ferroptosis, the GSH-dependent GPx4 pathway, and the NADPH-dependent FSP1 pathway. Glutamine (Gln); glutamate (Glu); cysteine (Cys); glycine (Gly); glutathione-disulfide reductase (GSR); stimulating proteins 1 and 3 (SP1/3); nuclear factor Y (NF-Y); cAMP-response element modulator-tau (CREM-tau); early growth response protein 1 (EGR1); nuclear factor κB (NF-κB); sterol regulatory-binding element 1 (SREBP1).

GPx4, a selenocysteine-containing, and glutathione-dependent enzyme, catalyzes the reduction of specific lipid hydroperoxides into lipid alcohols.^83^ GPx4 belongs to the family of Glutathione peroxidases (GPxs),^84^ but in contrast to other GPxs, GPx4 lacks a dimerization interface and exists as a monomeric species.^85^ GPx4 is a multifunctional protein capable of reducing peroxidized lipids either in free form or in complex with lipids such as PLs, with proteins such as lipoproteins or within membranes.^86^ This characteristic of reducing lipid peroxidation within membrane lipids determines its predominant role in preventing ferroptosis (Fig. 3). This catalytic reaction of GPx4 follows a ping-pong mechanism, whereby the enzyme active site shuttles between an oxidized and reduced state. First, the active site selenolate (Se–H) in GPx4 is oxidized to selenic acid (Se–OH) by a peroxide substrate. Then, the first glutathione (GSH) is used to reduce the selenic acid-generating an intermolecular selenylsulfide bond, which is reduced by a second GSH to form oxidized glutathione (GSSG) and regenerate the enzyme^87^ (Fig. 3).

GPx4 is also involved in the development and maintenance of a variety of physiological functions,^88^ and its genetic ablation or expression of an inactive form causes early embryonic lethality.^89^ However, disruption of mitochondrial GPx4 in mice is not lethal and causes infertility in males through abnormal sperm development.^90^ Neurons-specific deletion of GPx4 is neonatally lethal,^91^ while conditional GPx4 deletion in adult mice results in mitochondrial damage, hippocampal neurodegeneration, and increases astrogliosis.^92^ The ablation of GPx4 can trigger lipid-oxidation-induced acute renal failure and associated death.^80^

*GPx4 regulation—GSH axis*: GSH is essential for the GPx4-catalyzed reaction as it serves as an electron donor for reducing toxic phospholipid hydroperoxides (such as PE-AA-OOH/PE-AdA-OOH) to nontoxic phospholipid alcohols (such as PE-AA-OH, PE-AdA-OH), and the GSSG is generated as a by-product.^87^ GSH can be regenerated by reducing GSSG using glutathione reductase (GR). During this progress, reduced NADPH acts as the electron donor.^5^ NADPH abundance may be used as a predictor for the outcome of a ferroptotic event.^93^ Meanwhile, the regulation of cytosolic NADPH levels via Metazoan SpoT Homologue 1 (MESH1) can control ferroptosis.^94^ Therefore, GSH is considered as a critical factor for maintaining GPx4 activity.

GSH is synthesized from glutamate, cysteine, and glycine in two steps under the catalysis of the cytosolic enzymes glutamate-cysteine ligase (GCL) and glutathione synthetase (GSS) to participate in the regulation of ferroptosis^5^ (Fig. 3). Cysteine is the most limiting amino acid for GSH synthesis and inhibition of its import through the system ${\mathrm{X}}_c^-$ is sufficient to trigger ferroptosis in vitro.^6^ System ${\mathrm{X}}_c^-$ is a cystine/glutamate antiporter that facilitates the exchange of cystine and glutamate across the plasma membrane.^1,15^ Upon transport inside the cell, cystine is reduced to cysteine by GSH or thioredoxin reductase 1 (TXNRD1).^95^ Cysteine is a semi essential amino acid as it can be produced from methionine through the transsulfuration pathway converting methionine to homocysteine, cystathionine, and in a final step to cysteine.^96^ It has been shown that hepatocellular carcinoma cells are resistant to sorafenib, a novel multi-targeted oral drug for the treatment of tumors,^97^ which is acquired through transcriptional regulation of genes involved in lipid ROS and iron metabolism.^98^ Recently, inhibition of DJ-1 (also known as PARK7-parkinsonism-associated deglycase) has been shown to enhance the sensitivity of tumor cells to ferroptosis induced by sorafenib and evaluated the antitumor activity of erastin in a xenograft nude mouse model via inhibition of the transsulfuration pathway and limiting the supply of cysteine.^99^ P53, a tumor suppressor gene, is mutated in at least 80% of the most difficult-to-treat cancers, such as high-grade serous ovarian cancers, or triple-negative breast cancers.^100^ Cell-cycle arrest, apoptosis, and senescence are widely accepted as the major mechanisms by which p53 inhibits tumor formation. P53(3KR), an acetylation-defective mutant that fails to induce the above process, retains the ability to suppression of early-onset spontaneous tumorigenesis.^101^ The function of p53(3KR) tumorigenesis suppression is realized by inhibiting SLC7A11 expression and inducing ferroptosis in xenograft models.^102^ Similarly, BRCA1 associated protein 1 (BAP1) and OTU deubiquitinase, ubiquitin aldehyde-binding 1 (OTUB1), both involved in tumor regulation, can control ferroptosis via regulating the expression of system ${\mathrm{X}}_c^ -$.^103,104^ These facts indicate that the modulation of ferroptosis may be involved in the resistance of tumor cells to cell death and that system ${\mathrm{X}}_c^ -$ plays a central role in this process.

*GPx4 Regulation—Selenium axis*: Selenium is indispensable for mammalian life, and the deficiency of the selenocysteine- (Sec-) -specific tRNA gene Trsp (nuclear-encoded tRNA selenocysteine 2) is embryonic lethal.^105^ Selenium has been involved in many biological processes such as cancer prevention and promotion, protecting against cardiovascular diseases, and treating certain muscle disorders.^106^ The GPx4 mRNA contains a selenocysteine insertion sequence element in the 3’ untranslated region that encodes an active site of selenocysteine via a UGA codon.^107^ It has been suggested that selenium can regulate GPx4 expression through increasing ribosome density in downstream of UGA-Sec codons and sec incorporation efficiency in part by the degree of Sec-tRNA[Ser]SecUm34 methylation.^108^ The Sec-tRNA must first be activated by the addition of an isopentenyl lipid group, a product of the mevalonate (MVA) pathway.^109,110^ This may explain how disruption of the MVA pathway by statins leads to reduced GPx4 expression and increased ferroptosis in certain cells.^111^

The catalytic function of selenocysteine is due to its rapid deprotonation,^87^ while thiol groups remain protonated at neutral pH.^88^ It has been shown that under selenium deficiency conditions, cysteine can replace selenocysteine in various selenoproteins.^112,113^ However, replacing selenocysteine by cysteine in recombinant GPx4 leads to a 1000-fold reduction in catalytic activity.^114^ These facts suggest that selenocysteine as the catalytic moiety is necessary to guarantee a rapid reduction of hydroperoxide by GPx4 and prevent ferroptosis.^86^

#### The FSP1 pathway

FSP1 was primal disclosed as a p53‐responsive gene (PRG) in p53-mediated apoptosis, designated as PRG3. Because of the similarity in amino acid sequences between it and the human apoptosis-inducing factor (AIF), it was also named apoptosis-inducing factor (AIF)-like mitochondrion-associated inducer of death (AMID)^115,116^ or apoptosis-inducing factor mitochondria-associated 2 (AIFM2).^117^ Unlike AIF, FSP1 is predominantly cytosolic and may have an affinity for the cytosolic surface of the mitochondrial outer membrane. This is consistent with the apparent lack of an extended N-terminal mitochondrial targeting sequence analogous to that found in AIF.^116^ FSP1 was then found to be a flavoprotein oxidoreductase, and its proapoptotic function may be through inhibiting the redox activity. FSP1 can combine with DNA, nicotinamide coenzyme, and the modified flavin 6-hydroxy-FAD. When it binds to dsDNA (e.g., bacterial and (retro)viral), its function as an oxidoreductase will be antagonistic, leading to the accumulation of ROS.^117^ Doxorubicin treatments significantly enhance cardiac levels of 4-HNE and FSP1, and the 4-HNE adduction of FSP1 facilitates its translocation from mitochondria, which can change the activity of FSP1 to a proapoptotic protein.^118^ However, it has been shown that the expression of FSP1 induces cellular apoptosis at much lower levels than AIF in several different cell lines.^119^ A study has previously described AIF and FSP1 as type 2 NADH ubiquinone oxidoreductase (NDH-2) enzymes in mammalian mitochondria, reporting the possible interaction between FSP1 and ubiquinone.^120^ However, FSP1 lacks a mitochondrial localization sequence in AIF, so the role of FSP1 in cell death remains unclear.

Several studies have suggested that the sensitivity to GPx4 inhibitors varies greatly across different cell lines in ferroptosis.^121^ To explore the possible additional regulatory pathways, CRISPR/Cas9-based screens have been performed, and revealed that FSP1 is a previously unrecognized anti-ferroptotic gene.^2,23^ FSP1 catalyzes the regeneration of non-mitochondrial CoQ10 using NAD(P)H to block ferroptosis by inhibiting the propagation of lipid peroxides.^2,23^ Interestingly, membrane targeting of FSP1 via the myristoylation motif of N terminus is essential for its anti-ferroptotic activity. And selectively targetting FSP1(G2A), a mutant that lacks the myristoylation site, to the endoplasmic reticulum did not impact ferroptosis sensitivity.^2^ Indeed, it has been reported that the supplementation of cells with CoQ10 effectively suppresses ferroptosis as early as 2016,^122^ without a detailed mechanism.

CoQ10, as the main effector of the FSP1 pathway, is widely distributed among membranes of mammalian cells.^123^ It is composed of a benzoquinone ring and a polyisoprenoid tail containing between 6 and 10 subunits that are species-specific.^124^ Non-mitochondrial CoQ10 plays an essential role as a reversible redox carrier in the plasma membrane and Golgi apparatus membrane electron transport,^125^ and represents an essential endogenous lipid-soluble antioxidant by directly scavenging lipid peroxyl radicals.^126^ CoQ10 can reduce lipid hydroperoxides more effectively than α-toc.^127^ Moreover, oral administration of CoQ10 is reported in the treatment of various human diseases such as cardiomyopathy,^128^ Parkinson’s disease,^129^ and diabetes.^130^ In contrast, low CoQ10 levels due to mutations in CoQ10 biosynthetic enzymes or associated enzymes are associated with several diseases.^131–133^

CoQ10 biosynthesis pathway is tightly regulated both at the transcriptional and translational levels. CoQ10 can be synthesized using acetyl-CoA via MVA pathway^134^ (Fig. 3). MVA pathway enzymes condense three acetyl-CoA molecules in a two-step reaction to produce 3-hydroxy-3-methylglutaryl CoA (HMG-CoA). Then HMG-CoA reductase (HMGCR) reduces HMG-CoA to MVA via an irreversible reaction.^135^ MVA is then converted into isopentenyl-diphosphate (IPP) through a series of enzymatic steps, which serves as a monomeric unit for the consequent synthesis of all downstream metabolites.^136^ IPP is catalyzed into CoQ10 through various enzymatic steps,^137^ including two major intermediates, farnesyl diphosphate (FPP) and geranylgeranyl-diphosphate (GGPP).^138^

#### Other antioxidant pathways

In addition to NAD(P)H-FSP1-CoQ10 and NAD(P)H-GSH-GPx4 as two parallel pathways that suppress ferroptosis, other natural antioxidants can also play a part in preventing ferroptosis, such as vitamin E,^139^ thioredoxin,^140^ and mitoquinone.^141^ Recent research discovered that inducible nitric oxide synthase (iNOS)/NO• abundance modulates susceptibility to ferroptosis in macrophages/microglia.^78^ BH4 is a potent radical-trapping antioxidant that protects cells from ferroptosis upon GPX4 inhibition by reducing lipid peroxidation and is regenerated by dihydrofolate reductase (DHFR).^142^ Besides, nuclear factor erythroid 2-related factor 2 (Nrf2) may play a role in modulating the cellular ferroptosis response. Nrf2 is responsible for regulating several antioxidant genes.^143^ Importantly, almost all genes implicated in ferroptosis are transcriptionally regulated by Nrf2, including genes of glutathione regulation, NADPH regeneration, and iron regulation.^144–147^ Moreover, Nrf2 indirectly modulates the lipids, whose abundance contributes to ferroptosis sensitivity.^6,148^ Consequently, Nrf2 activation results in resistance to ferroptosis in cancer cells,^98,149^ and other cell types.^150^

### The link between ferroptosis and other cell death pathways

#### Oxytosis

Oxytosis was introduced as a form of non-apoptotic regulated cell death in 2001, which was characterized by oxidative stress and GSH depletion.^151^ In the early studies, oxidative glutamate toxicity served as a specific example of the more general oxytosis pathway. The link between glutamate toxicity and GSH depletion was established by the glutamate-mediated inhibition of cystine uptake by the system ${\mathrm{X}}_c^ -$.^16^ GSH depletion during oxytosis can be expected to impair GPx4 activity as GSH is required for GPx4 activity.^152^ Most of the oxytosis studies have been carried out in HT22 cells that were explicitly sensitive to glutamate toxicity.^91^ The defective GPx4 expression can enhance cytotoxicity by glutamate-induced oxytosis in the retina.^153^ Interestingly, the lipophilic antioxidant α-toc can efficiently inhibit oxytosis.^154^ Exogenous AA potentiates oxytotic cell death, while multiple Lox inhibitors can protect from GSH depletion.^91^

The characteristics of oxytosis are consistent with ferroptosis. Oxytosis in HT22 cells can be inhibited by iron chelators and exacerbated by different sources of iron.^155^ Calcium entry into cells is a necessary step in oxytosis.^151^ Glutamate induces a significant increase in intracellular Ca^2+^ about 30–50 fold,^156^ and inhibitors of calcium entry could effectively inhibit the occurrence of oxytosis.^151^ However, the role of calcium in ferroptosis has not been established. Both oxytosis and ferroptosis induce the expression of eIF2α.^151,157^ Bid knockout using CRISPR/Cas9 approaches can protect neurons against both ferroptosis and oxytosis.^47^ The pharmacological inhibition of double-stranded RNA-dependent protein kinase-mediated neuroprotective effects against both ferroptosis and oxytosis.^158^

#### Autophagic cell death

The process that removes intracellular components such as unused proteins and damaged organelles through lysosomes, was named “autophagy” in 1963.^159^ Autophagy plays a multifaceted role in regulating both the quality and quantity of proteins and organelles,^160^ therefore it determines cell fate via various pathways.^161–163^ Recent studies have placed the autophagy process in ferroptosis since it regulates the abundance of ferritin, the major iron storage protein. NCOA4 is a selective cargo receptor for the autophagic turnover of ferritin by lysosomes.^67^ Genetic and pharmacological inhibition of NCOA4 can protect cells from ferroptosis via reducing cellular labile iron.^9^ Inhibition of lysosomal function, the endpoint of autophagy flux, can significantly block erastin-induced ferroptosis in both MEFs and HT1080 cells.^9^ Dihydroartemisinin also promotes ferroptosis by inducing ferritinophagy and increasing the labile iron pool in acute myeloid leukemia.^164^

Similarly, lipophagy, which is a form of selective autophagy that leads to the autophagic degradation of intracellular lipid droplets (LDs), also can regulate ferroptosis.^165^ The knockdown of the LD cargo receptor RAB7A can inhibit ferroptosis.^166^ In contrast, the overexpression of TPD52 (tumor protein D52) limits RSL3-induced ferroptosis by increasing lipid storage.^167^ These studies have strengthened the link between autophagy and ferroptosis.

In Tables 1 and 2, we summarized some of the currently used small molecules and drugs that interfere with ferroptosis, the postulated mechanism and the corresponding cellular/animal experimental model.

**Table 1:** Summary of ferroptosis inducers.

Compound/drug	Target	Mechanism	Model	References
Erastin	System X_*c*_−	Interfere cystine uptake and deplete GSH, increase LIP level	Cell line: HT-1080, SH-SY5Y	^1,290^
Piperazine erastin	System X_*c*_−	Upregulate PTGS2, suppressed by vitamin E	BJeLR cells	^13^
Imidazole ketone erastin	System X_*c*_−	Interfere cystine uptake and deplete GSH	Cell line: G-401, DLBCL xenograft model	^38,184^
Sulfasalazine	System X_*c*_−	Interfere cystine uptake and deplete GSH	Nb2 lymphoma cells	^291^
Sorafenib	System X_*c*_−	Blocks system X_*c*_− and deplete GSH	HCC cells	^292^
Glutamate	System X_*c*_−	Interfere cystine uptake and deplete GSH	HT-1080 cells	^293^
BSO (buthionine sulfoximine)	Glutamate-cysteine ligase	Mediate glutathione deficiency	Newborn rats	^294^
DPI2		Interfere cystine uptake and deplete GSH	BJeLR cells	^13^
Cyst(e)inase	Cysteine consumption	Deplete L-Cysteine via interfering transsulfuration pathway and/or increasing ROS production	PCa cells, FVB/N mice	^191^
BAY 87-2243	Mitochondrial complex I	Suppress the activity of Mitochondrial complex I, increase ROS	Cell line: H460, G361 and SK-MEL-28	^295,296^
Artesunate	Nrf2− antioxidant response element	Downregulate GSH level, upregulate lipid ROS and mediate ferritinophagy	Cell line: HNC, LX-2; ICR mice,	^149,297^
(1S,3R)-RSL3	GPX4	Inhibit the activity of GPX4 via binding selenocysteines at active-site	Cell line: BJeLR, HT-1080	^298^
ML162, ML210, DPI 7, DPI 10, DPI 12, DPI 13, DPI 17, DPI 18, DPI 19	GPX4	Inhibit the activity of GPX4	BJeLR cells	^13,299^
Altretamine	GPX4	Inhibit the activity of GPX4	U-2932 cells	^300^
Withaferin A	GPX4 and KEAP1 inactivation	Stimulate Nrf2 via binding KEAP1, inhibit GPX4	IMR-32 and SK-N-SH cells	^172^
FIN56	GPX4 and squalene synthase	Increase degradation of GPX4, suppress CoQ10 via targeting and stimulating SQS	Cell line: BJeLR, HT-1080, PACN1, MEFs	^122,301^
Statins (fluvastatin, lovastatin, simvastatin)	HMGCR	Inhibit HMGCR and suppress GPX4 biosynthesis	Cell line: HT-1080, HCC4006	^111,122^
Hemoglobin		Release iron and produce lethal ROS	Cell line: OHSCs	^302^
Hemin		Cause high level of HMOX1 and increase intracellular iron	Cell line: IMR-32, HT22, primary cortical neurons; Male Swiss albino mice	^172,303^
FeCl2, (NH4)2Fe(SO4)2		Release iron and produce lethal ROS	Cell line: IMR-32, OHSCs	^172,302^
Non-thermal plasma	Ferritin	Break ferritin and induce reduction from Fe(III) to Fe(II)	Cell line: IMR-90-SV, SAS, Ca9-22	^169^
Salinomycin, ironomycin	DMT1, ferritin, GPX4	Decrease expression of GPX4 and ferritin, and inhibit DMT1 by interrupting lysosomal iron translocation	Cell line: BCSCs, CSC	^171,206,304^
Siramesine + lapatinib	Iron transport	Increase transferrin and decrease ferroportin	Cell line: MDA MB 231, MCF-7, ZR-75, SKBr3, A549, U87	^305,306^
FINO2 (1,2-dioxolane)	Lipid	Inactivate GPX4 and lead to Fe(III) oxidation	Cell line: IGROV-1, NCI-H322 M, NCI60, BJ-hTERT	^170,307^
BAY 11-7085	IκBα	Increase HO-1 related to redox regulation	Cell line: MCF-7, MDA-MB-231, MDA-MB-468, SKBR3	^308^
Trigonelline,brusatol	NRF2	Inhibit Nrf2	Cell line: HNC, HNSCC	^149,309^
Artemisinin derivatives		Induce ROS and mediate oxidative stress	CCRF-CEM cells	^310^
CIL41, CIL56, CIL69, CIL70, CIL75, CIL79		Induce ROS(CIL56 mediate ferroptosis at low concentration while necrotic, non-suppressible phenotype at high)	Cell line:BJ cells, HT-1080	^122^

**Table 2:** Summary of ferroptosis inhibitors.

Compound/drug	Target	Mechanism	Model	References
Vitamin E, α-toc, trolox, tocotrienols	LOX	Restrain LOX PUFA oxygenation	Cell line: PBMCs, Pfal1; Gpx4 KO C57BL/6J mice	^11,26^
Deuterated polyunsaturated fatty acid	Lipid peroxidation	Inhibit lipid peroxidation	APP/PS1 mice	^232,311^
Butylated hydroxytoluene, butylated hydroxyanisole	Lipid peroxidation	Inhibit lipid peroxidation	C57BL/6J mice	^312,313^
Ferrostatins, liproxstatins	Lipid peroxidation	Inhibit lipid peroxidation	Cell line: HEK-29, HT22, HT-1080	^1,20^
CoQ10, idebenone	Lipid peroxidation	Target lipid peroxyl radicals	Cell line: HT1080, Pfa1, NCI-H460, NCI-H2291, NCI-H1703 and NCI-H446	^2,23^
XJB-5-131, JP4-039	Lipid peroxidation	Nitroxide-based mitochondrial lipid peroxidation mitigators	Cell line: HT-1080, BJeLR, and panc-1 cells	^314^
Baicalein	LOX	Inhibit 12/15-LOX	HT22 cells, TBI mice model	^315,316^
PD-146176	LOX	Inhibit 15-LOX-1	HEK-293 cells	^20^
AA-861	LOX	Inhibit 5-LOX	HEK-293T cells; ALF rat	^317,318^
Zileuton	LOX	Inhibit 5-LOX	Cell line: LNCaP, K562, HT22	^53,319^
Deferoxamine, ciclopirox, deferiprone	Iron	Reduce intracellular iron	HT-1080	^1^
Glutamine deprivation, glutaminolysis inhibitor	Glutaminolysis	Maybe hinder mitochondrial TCA cycle	Cell line: HT-1080, MEFs	^19,48^
Cycloheximide	Protein synthesis	Inhibit xCT protein synthesis	Primary cortical neurons	^320^
β-mercaptoethanol	Reducing agent	Reduce Cys2 to Cys	OT-1 CD8þ T cell	^321^
Dopamine	Neurotransmitter	Increase the stability of GPX4	Cell line: PANC1, HEY, MEF, HEK293	^322^
Selenium	Selenoproteins	Enhance the number of selenoproteins	Cell line: MEFs, HT-1080	^22,247^
Vildagliptin, alogliptin, linagliptin	Dipeptidyl-peptidase-4	Reduce lipid peroxidation via inhibiting DPP4	TP53-deficient CRC cells	^323^

## Links between ferroptosis and disease

### Cancer

Cancer cells accumulate high levels of iron as compared to normal cells.^168^ Research has advocated the abnormality of iron homeostasis in several cancer types, including breast cancer, ovarian cancer, renal cancer, and lung cancer.^62^ Non-thermal plasma (NTP) breaks ferritin and induces reduction from Fe^3+^ to Fe^2+^, accompanied by lipid peroxidation and mitochondrial superoxide generation, which selectively eliminates oral squamous cell carcinoma cells.^169^ FINO2, an endoperoxide-containing 1,2-dioxolane, can oxidize Fe^2+^ leading to lipid peroxidation and kill BJeLR cancer cells via ferroptosis.^170^ Silencing the expression of prominin2 decreases the cellular iron export of RSL3-treated mammary epithelial (MCF10A cells) and breast carcinoma cells (Hs578t cells) via reducing the formation of ferritin-containing multivesicular bodies (MVBs).^68^ Inhibition of NFS1, a [Fe–S] cluster biosynthetic enzyme, stimulates the expression of transferrin receptor but restrains ferritin, causing iron-starvation response and leading to ferroptosis in lung cancer cells.^8^ Salinomycin has been reported to kill cancer stem cells in a mechanism involving iron sequestration within lysosomes, leading to ferroptosis and lysosomal membrane permeabilization.^171^ In high-risk neuroblastoma, withaferin A (WA) blocking the function of Kelch-like ECH-associated protein 1 (KEAP1) can reduce the inhibition of Nrf2 that indirectly increases LIP through heme oxygenase-1 (HO-1) and kills tumors via the KEAP1-Nrf2 pathway, a noncanonical pathway of ferroptosis.^172^

Cancer cells need a high metabolic rate to maintain their rapid proliferation, accompanied by an increase in ROS production.^173^ Therefore, high ROS levels are an inherent feature of tumors, and cancer cells have to boost their antioxidant defense capacity to overcome this enhanced oxidative stress.^174^ Targeting the antioxidant defense mechanism of cancer cells may be an effective potential treatment strategy by predisposing them to oxidative stress-induced cell death, such as apoptosis and ferroptosis. The cytotoxicity induced by chemotherapeutic drugs such as 5-FU, oxaliplatin, and paclitaxel is linked with elevated ROS,^175–177^ and depletion of intracellular GSH using RNAi against the anti-oxidant transcription factor Nrf2 leads to increased ROS and increased sensitivity to chemotherapy in preclinical studies.^178^ ROS initiate the oxidation of PUFAs and play an important role in non-enzymatic lipid peroxidation or auto-oxidation of lipids.^32^ In several arsenic trioxide-resistant human leukemic cell lines, the DHA can enhance the cytotoxic effect of As2O3 through an increase of intracellular lipid peroxidation products.^179^ WA inactivates GPx4 and induces ferroptosis via accumulating lipid peroxides to toxic levels, which might also explain its ability to kill high-risk neuroblastoma cells and inhibit tumor growth of neuroblastoma xenografts.^172^ Interestingly, peroxisomes may contribute to ferroptosis through synthesizing PUFA-ePLs, and 786-O tumor xenografts can evade GPx4 knockout induced ferroptosis in mice by downregulating PUFA-ePLs.^24^ These facts highlight the potential value of lipid peroxidation and ferroptosis in tumor suppression strategies.

Ferroptosis is suggested to be a good approach to circumvent the therapy resistance of cancer cells. Once ‘epithelial-to-mesenchymal’ transition (EMT) occurs in tumor cells, which will acquire drug resistance and be intractable for treatment. By gene-signature, proteomic, and lineage-based correlation analyses, therapy-resistant high mesenchymal state cancer cells are found to be dependent on GPx4 for their survival and thus vulnerable to ferroptosis.^111^ ZEB1, which promotes tumor invasion and therapy-resistant by inducing EMT in carcinoma cells, was found to regulate the sensitivity of mesenchymal state cancer cells to GPx4 inhibition as a lipogenic factor.^111^ In a recent study, the loss of tumor suppressor Merlin, a frequent tumourigenic event in mesothelioma, dictates GPx4 dependency in murine models of mesothelioma through the upregulation of multiple ferroptosis modulators, including ACSL4 and transferrin receptor.^54^ Coincidentally, the residual persister cancer cells, which contribute to tumor relapse, acquire a dependency on GPx4 to survive.^180^ In these tumor cells, the antioxidant genes, such as NADPH, GSH, and Nrf2, were significantly downregulated, so the inhibition of GPx4 results in selective persister cell ferroptotic death in vitro and prevents tumor relapse in vivo.^180^ Melanoma usually metastases to lymph nodes before forming distant metastases due to the low cell survival rate in the blood, which is one of the most important predictors of distant metastasis and death in patients with cutaneous melanomas.^181,182^ It is recently shown that the lymphatic environment protects metastasizing melanoma cells from ferroptosis to increase their survival rate during subsequent metastasis through blood.^183^ By pretreating with ferroptosis inhibitors, the melanoma cells can form more metastases than untreated cells after intravenous injection in immunocompetent mice.^183^ In addition, pharmacological and genetic regulation of GSH can also effectively kill tumor cells. Imidazole ketone erastin (IKE) can alleviate tumor growth via interfering with cystine uptake in a diffuse large B cell lymphoma (DLBCL) xenograft model.^184^ Combinational treatment with ferroptosis inducing drugs with vemurafenib, a BRAF kinase inhibitor, results in a substantial decrease in long-term persisting melanoma cell.^185^

Furthermore, ferroptosis may participate in tumor immunity. Dendritic cells (DCs) appear essential for antitumor immunity via conditioning the tumor microenvironment and regulating priming of antitumor T cells.^186^ It has been reported that ALox15-derived lipid peroxide regulates DCs maturation and modulates adaptive immune responses.^150^ Oxidized phosphatidylcholine inhibited DC maturation via the activation of the transcription factor Nrf2 and dampened the differentiation of T helper 17 (TH17) cells.^150^ Similarly, oxidized phosphatidylethanolamines are also involved in the immune response, which can clear apoptotic cells by mouse inflammatory macrophages in vitro and in vivo.^187^ IFNγ plays an essential role in antitumor immune response, and Mauguso et al. confirmed that resistance to immunotherapy is attributed to defects in IFNγ signaling.^188^ Interestingly, IFN-γ can suppress the expression of SLC7A11A and SLC3A2, two essential proteins for the synthesis of GSH, leading to the transport deficit of cystine, which lipid peroxidation and ferroptosis in cancer.^189^

Adaptive immunity is an indispensable and powerful ‘weapon’ in tumor immunity, and ferroptosis may also be involved in adaptive immunity.^190^ In T cell-specific GPx4-deficient mice, healthy thymic T cell development and T cell responses upon secondary infection were observed. However, both antigen-specific CD8^+^ and CD4^+^ T cells failed to expand and to protect from acute infections, and CD8^+^ T cells had an intrinsic defect in maintaining homeostatic balance in the periphery,^190^ which can be rescued with a high dosage of vitamin E.^190^ Ferroptosis also plays a significant role in CD8^+^ T cell-induced cancer cell death in cancer immunotherapy.^189^ Mechanistically, interferon-gamma (IFNγ) from CD8^+^ T cells triggers lipid peroxidation and ferroptosis by the inhibition of system ${\mathrm{X}}_c^ -$.^189^ Cyst(e)inase, an engineered enzyme that degrades cystine, efficiently induces oxidative stress and ferroptosis. A combination of cyst(e)inase and PD-L1 can strongly inhibit the growth of ID8 cell-derived tumors,^191^ accompanied by increased lipid peroxidation in tumor cells and increased percentages of IFNγ^+^ and TNF^+^ CD8^+^ and CD4^+^ T cells in the tumor microenvironment.^189^ Notably, In cancer patients, the expression of system ${\mathrm{X}}_c^ -$ was negatively associated with CD8^+^ T cell signature, IFNγ expression, and patient outcome.^189^ In humoral immunity, GPx4 is essential to prevent ferroptosis during development, maintenance, and responses of innate-like B cells.^192^ However, in germinal center reactions, and antibody responses of follicular B2 cells, it is not GPx4-dependent.^192^ Therefore, the synergy of ferroptosis and tumor immunotherapy may be a potential cancer treatment strategy.

Ferroptosis is involved in many anti-tumor therapies, including radiotherapy, one of the standard methods used in clinical cancer treatment. The antitumor effects of irradiation are attributed to the microparticles released by irradiated cells, which were shown to induce immunogenic death mainly through ferroptosis.^193^ The expression of the Ataxia-Telangiectasia mutated gene in irradiated tumor cells would inhibit the expression of SLC7A11, blocking cystine uptake and resulting in lipid peroxidation accumulation.^194^ Ionizing radiation also upregulates the expression of ACSL4 in human cancer cells.^55^ Erastin treatment in HeLa and NCI-H1975 adenocarcinoma cell lines aggravates radiation-induced cell death.^195^ Clinical drugs, such as sorafenib,^196^ sulfasalazine,^197^ artemisinin,^198^ and ibuprofen,^199^ may induce ferroptosis in cancer cells, and ferroptosis inducers combined with temozolomide, cisplatin, haloperidol, and doxorubicin can enhance the chemotherapy effect of these drugs in the treatment of tumors.^200–202^ There are to date numerous studies on inducing ferroptosis in cancer cells by increasing lipid peroxidation; however, they remain in preclinical stages.

Over the past decade, nanotechnology has made significant contributions to oncology, and it has great potential in cancer treatment. Several studies have applied nanotechnology to induce ferroptosis for the development of cancer therapies. The first ferroptosis-inducing nanoparticles were described in 2016, where the authors have shown that intravenous injection of ultrasmall poly(ethylene glycol) (PEG)-coated silica nanoparticles can reduce the growth of the tumor by inducing ferroptosis in mice.^203^ In 2018, FeGd-HN@Pt@LF/RGD2 nanoparticles that deliver Fe^2+^ and Fe^3+^ were used for the treatment of orthotopic brain tumors.^204^ In 2019, up-conversion nanoparticles (UCNP) that can release Fe^2+^ and induce ferroptosis had been invented and tested in 4T1 xenograft mice.^205^ Subsequent studies have used several different strategies to induce ferroptosis in cancer cells, including particles called salinomycin-loaded gold nanoparticles(AuNPs), which leads to iron accumulation and intracellular GSH exhaustion,^206^ a lipid peroxidation generator consisting of a novel GSH and iron redox couple^207^ and oxygen-boosted phototherapy.^208^

### Neurodegeneration

The growing incidence of neurodegenerative diseases has brought a considerable burden to society and significant distress to both patients and caregivers. However, there are still limited treatment strategies for these diseases.^209^ Therefore, it is urgent to impel further exploration of the relationship between pathological characteristics, disease mechanism, and neuronal death. Here, we mainly focus on the relationship of ferroptosis with Alzheimer’s disease (AD) and Parkinson’s disease (PD).

Iron dyshomeostasis and lipid peroxidation, hallmarks of ferroptosis, have long been noted in AD and PD pathology. Aging is the major risk factor for neurodegenerative diseases and is accompanied by brain iron accumulation.^210^ Similarly, iron accumulation in affected brain regions of diseases has been reported in various neurodegenerative diseases.^211–213^ Thus, iron has been suggested as an essential factor contributing to the neurodegenerative processes. Iron can lead to the dissociation of IRPs from the IRE by binding to IRPs, altered translation of target transcripts.^214^ Interesting, IREs are found in the 5’-UTR of amyloid precursor protein (APP) and α-synuclein (α-Syn) transcripts, and iron accumulation can upregulate the levels of α-Syn, APP and amyloid β-peptide (Aβ).^215^ And deferoxamine (DFO), as a widely used iron chelator, can inhibit amyloidogenic APP processing and Aβ aggregation in animal studies.^216,217^ In separate randomized controlled trials, deferiprone (DFP), an orally bioavailable brain permeable iron chelator, was shown to alleviate nerve function scores and ameliorate iron-related neurological symptoms.^218–220^ Similarly, DFP has reported beneficial effects for PD patients in phase II studies.^221,222^ The loss of ceruloplasmin (Cp), a protein responsible for iron export, is also associated with iron-dependent parkinsonism in mice,^223^ and in the cerebrospinal fluid (CSF) of PD patients.^224^ As discussed earlier, iron is also involved in ROS production and lipid peroxidation, and the iron accumulation is always accompanied by oxidative stress.

Due to its high metabolic activity, brain tissues are particularly vulnerable to oxidative stress.^225^ Increased oxidative stress is a feature of several neurodegenerative diseases, including Alzheimer’s disease^226^ and Parkinson’s disease.^227^ Piceid, as a natural antioxidant, can protect the vulnerable SNc neurodegeneration via correcting several major anti-oxidant pathways/parameters, including GSH, MDA and the SOD, selectively in three rodent models of PD.^228^ Similarly, the use of antioxidants, such as MitoQ and SOD2, can alleviate the pathological characteristics in AD animal models.^229,230^ Furthermore, neuronal membranes are rich in PUFAs, that are prone to oxidation,^231^ and consequently, lipid peroxidation is likely to contribute to oxidative stress associated with neurodegeneration. Isotope-reinforced (deuterated) PUFA (D-PUFA) is effective in reducing lipid peroxidation and Aβ level in the APP/PS1 transgenic mouse model of Alzheimer’s disease.^232^ Alpha-Lipoic acid (ALA), a fat-soluble and water-soluble antioxidant and also a naturally occurring enzyme cofactor with reducing lipid peroxidation properties, can significantly alleviate AD pathology in P301S tau transgenic mice with alleviated properties of ferroptosis.^233^ Similarly, 2000 IU/d of α-toc compared with placebo resulted in a slower functional decline among patients with mild to moderate AD.^234^

Copper(II)-diacetyl-bis(4-methylthiosemicarbazonato) (Cu-ATSM) is a PET tracer initially developed for hypoxia imaging but has recently shown neuronal protection in multiple PD models and prevent lipid peroxidation without altering the oxidation state of iron.^235^ Cu-ATSM obtains the anti-ferroptotic activity like liproxstatin-1 by preventing the propagation of lipid radicals rather than preventing iron oxidation. Combined with its ability to enter the brain, Cu-ATSM may be an attractive investigational product for clinical trials of ferroptosis and neurodegeneration.^236^ The maintenance of glutathione GSH is a key antioxidant element in brain redox homeostasis. In the AD model, N-acetyl cysteine (NAC) can protect neurons function and improving learning and memory deficits via increasing GSH levels along with the reduced MDA,^237^ which may also be related to the anti-amyloid efficacy of NAC.^238^ Nigral GSH loss and oxidative stress are predispositions to PD,^239^ and in a randomized, double-blind placebo-controlled clinical trial, the oral adjunction of omega-3 fatty acids and vitamin E for three months improves GSH level and Unified Parkinson’s Disease Rating Stage (UPDRS).^240^ In a recent epigenetic study on blood-based methylome-wide association study of PD, it has been identified that hypermethylation in the promoter region of the SLC7A11 gene can downregulate system ${\mathrm{X}}_c^-$ along with the reduced GSH synthesis and increased sensitivity to ferroptosis.^241^ Similarly, there is a linear correlation between GSH loss and neurodegeneration in neurons cultured from aged 3xTg-AD mice, and 3xTg-AD neurons are more dependent on GSH availability than the non-Tg neurons.^242^

These data implicate a potential link between ferroptosis and neurodegenerative diseases, while several subsequent studies have provided more direct evidence. In 2018, it was shown that tau overexpression and hyperphosphorylation can induce neuronal loss via ferroptosis, and ALA supplementation effectively inhibited cognitive decline through reducing tau-induced iron overload, lipid peroxidation, and upregulating GPx4 expression in P301S tau transgenic mice.^243^ Moreover, a targeted mutation of GPx4 (selenocysteine to cysteine substitution) or GPx4 conditional deletion in neurons causes neuronal toxicity and rapid neuronal death in mice, which are accompanied by multiple ferroptotic characteristics.^22,92^ GPx4 is also critical for maturation and survival of photoreceptor cells; photoreceptor-specific rapidly underwent severe degeneration and completely disappeared by conditional knock-out GPx4.^244^ Targeted conditional knockout of GPx4 in forebrain neurons of adult mice causes AD-like cognitive impairments and neurodegeneration that can be attenuated by ferroptosis inhibitors.^245^ Also, the expression of GPx4 might play a neuroprotective role in PD pathology.^246^ It is shown that GPx4 colocalizes with AS-positive nigral Lewy bodies and dystrophic TH-positive fibers in the putamen, and it is increased relative to cell density, probably because of an increase in survival of cells expressing GPx4.^246^ There is a correlation between selenium level, a key factor for GPx4 activity, and susceptibility to ferroptosis.^247^ In AD patients, the selenium level can be related to the pathological progress of the disease.^248,249^ The treatment of selenium attenuates a beta production by reduced 4-HNE-induced transcription of beta-secretase (BACE1) and protects against Aβ-mediated toxicity in primary cultured neurons.^250^ In a paraquat-induced rat PD model, selenium feeding also can reduce bradykinesia and DNA damage.^251^ Depletion of DJ-1, a known cause of early-onset autosomal recessive Parkinson’s disease,^252^ can also increase the sensitivity of neurons to ferroptosis by markedly reduce GSH levels through inhibition of the transsulfuration pathway.^99^

Besides, ferroptosis inhibitors are protective in cellular models of Huntington’s disease,^253^ and an MPTP mouse model of Parkinson’s disease.^254^ Recently promising results were reported for the use of an anti-ferroptotic compound in phase I clinical trials in amyotrophic lateral sclerosis (ALS) patients and PD patients.^236^ Therefore, ferroptosis may play an essential role in the pathogenesis of various neurodegenerative diseases, and anti-ferroptotic strategy should be further investigated.

### Ischemia/reperfusion

Ischemia/reperfusion (I/R) is a pathological condition contributing to morbidity and mortality in a wide range of conditions. When the tissue experiences obtunded blood flow by blockage or rupture of an artery, the ischemia occurs. The interruption of blood supply means the exhaustion of energy and cell death. So it is necessary to restore blood supply as soon as possible. However, more grave functional and structural changes become evident in the process of blood flow recovery.^255^ This pathological progress is ischemia/reperfusion injury(IRI), and it can trigger myocardial infarction, acute kidney injury, circulatory arrest, and even sleep apnea. However, IRI is also a significant challenge in organ transplantation,^256^ and the adverse effects of IRI in clinical situations are difficult to limit.

**Fig. 4:**
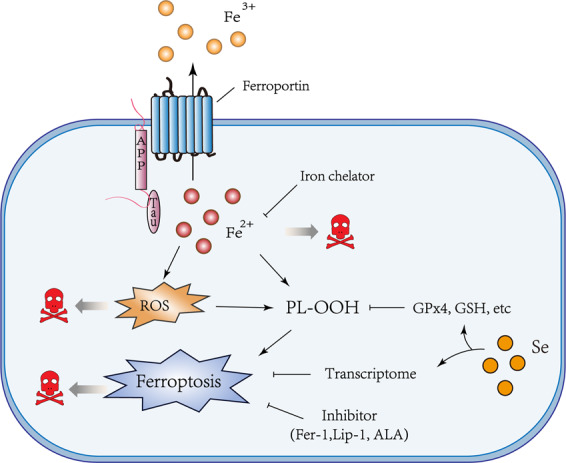
Iron, ROS, ferroptosis, and ischemia-reperfusion injury. The relationships between iron, ROS, and ferroptosis in ischemia/reperfusion has been illustrated, where ferroptosis may be a result of accumulated ROS, induced by impaired iron export. Iron chelators, anti-oxidants, and inhibitors of ferroptosis may prevent the toxic reaction.

Iron is a potential therapeutic target for IRI (Fig. 4). Clinical studies have shown that children with following severe ischemic-anoxic insult have significantly increased iron levels in multiple areas of the brain.^257^ Also, increased iron levels during I/R were proposed to mediate tissue damage in IRI.^258–262^ Supporting evidence was provided by reduced IRI damage following iron chelation in several animal models of IRI.^263–265^ It has also been proved that adjunctive DFO treatment can ameliorate oxidative stress injury in ST-elevation-myocardial infarction.^266^

In addition, it is well known that reperfusion of ischemic tissue can lead to a “burst” of ROS, which leads to further deterioration and tissue damage^267^ (Fig. 4). Accordingly, antioxidants were shown to protect from IRI in various conditions.^268–271^ It is also known that the increase in oxidation is also accompanied by lipid peroxidation^272^ (Fig. 4). ALA, as described before, can raise glutathione intracellularly, and it has a protective effect on ischemia-reperfusion injury in a variety of clinical conditions, such as simultaneous kidney-pancreas transplantation,^273^ human liver transplantation,^274^ and liver resection.^275^ The levels of lipid peroxide is markedly increased, while the levels of GSH and GPx4 are significantly reduced in rodent models of ischemic stroke, and carvacrol protected hippocampal neurons by increasing GPx4 expression.^276^ Knockout of GPx4 induces kidney failure in mice, which can be inhibited by lipid peroxidation inhibitors.^80^ Similarly, cardiac IRI can be alleviated by mitochondria-specific overexpression of GPx4.^277^

Intriguingly, the regulation of iron and lipid peroxidation can impact ferroptosis sensitivity, and accumulated evidence shows that the regulation of these two vital factors can affect IRI by controlling the ferroptosis (Fig. 4). I/R in the brain can acutely suppress tau expression, an Alzheimer’s disease protein that can facilitate iron export,^278,279^ causing iron accumulation accompanied by ferroptosis in the infarct zone, which aggravated neuronal damage.^262^ Iron chelation and ferrostatin-1 have similar protective effects on heart failure induced by both acute and chronic I/R.^280^ Pharmacological selenium significantly reduces infarct volume by driving an adaptive transcriptional program to block ferroptosis.^247^ The inhibition of glutaminolysis, involved in the NADPH-GSH-GPx4 pathway, can attenuate cardiac IRI by blocking ferroptosis.^19^

In addition, ACSL4 was upregulated in human ischemic intestinal tissues compared with that in healthy tissues, and liproxstatin-1 and siRNA to inhibit ischemia/hypoxia-induced ACSL4 ameliorated I/R-induced intestinal injury.^56^ 12/15-LOX knockout mice can protect neurons against cerebral ischemic injury.^281^ The heart after acute and chronic I/R is accompanied by severe cardiomyopathy. In DOX-treated murine hearts, HO-1 was significantly upregulated, promoting systemic accumulation of nonheme iron via heme degradation and accompanied by lipid peroxidation and ferroptosis.^280^ Ferrostatin-1 can significantly reduce DOX cardiomyopathy.^280^ And ferrostatin-1 also has been shown to ameliorate heart failure induced by I/R.^280^

Similarly, the inhibition of ferroptosis was effective in attenuating I/R-associated renal injury.^80,282^ Augmenter of liver regeneration (ALR) can also affect kidney injury by regulating ferroptosis in renal I/R.^283^ The inhibition of ALR using short hairpin RNA lentiviral (shRNA) aggravates pathology progression and leads to increased ROS, mitochondrial damage, and ferroptosis.^283^ Moreover, in intestinal IRI and testicular IRI models, the inhibition of ferroptosis produces a significant protective effect.^56,284^ These data reinforce the relevance of ferroptosis in I/R and present new avenues for using ferroptosis inhibition as a therapeutic strategy for I/R related damage.

### Other pathological conditions

Doxorubicin (DOX) is a commonly used chemotherapeutic drug for the treatment of breast cancer, leukemia, and other malignancies, but its use is limited by the severe toxic side effects, which may cause cardiomyopathy and heart failure.^285^ Ferroptosis inhibitors can protect against DOX-induced cardiomyopathy.^280^ Besides, ferroptosis is involved in other pathological conditions, such as hemochromatosis, cystic fibrosis, chronic obstructive pulmonary disease, and Pelizaeus–Merzbacher disease.^76,286–288^

## Concluding remarks

Ferroptosis research still faces its challenges as several mechanistic aspects of ferroptotic cell death are not well understood. Notably, the role of iron and lipoxygenases in triggering or propagating lipid peroxidation and the contribution of organelles such as mitochondria are under extensive investigation.

Ferroptosis lead to an imbalance of redox state and to a sequence of events different from other types of cell death, which includes iron liberation from ferritin and lipid peroxidation. However, why does this imbalance not simply trigger apoptosis? It was previously found that ferroptosis and necroptosis can be alternatives, in that necroptosis drives basal resistance to ferroptosis through depleting PUFAs and ferroptosis also drives basal resistance to necroptosis by reducing membrane permeabilization.^289^ It is yet to define whether ferroptosis is a specific mechanism to inhibit other death pathways.

Also, whether ferroptosis occurs as an automatic response to diverse stimuli that destabilize the metabolic balance, or it is the stimuli that directly disrupt the balance and cause ferroptosis. Is ferroptosis achieved “actively” or “passively”? Increasing evidence has shown the crosstalk between ferroptosis and other cell death. The further illumination of this interrelation also is a requisite for exploring the associated mechanisms and developing treatments.

Therefore, it is vital to selectively label cells undergoing ferroptosis, which will facilitate the exploration of the role of ferroptosis in pathological and physiological contexts. Most probes are limited on biochemical assays; thus, specific indicators to identify cells that are explicitly undergoing ferroptosis in tissue sections would greatly facilitate our understanding of ferroptosis and its potential therapeutic use in diseases.
